# Effects of Edible Grass (*Rumex patientia* L. × *Rumex tianschanicus* A. LOS) Leaf Powder on Growth Performance, Antioxidant Properties, Cecal Short-Chain Fatty Acids, and Microbial Community Levels in Broilers

**DOI:** 10.3390/antiox13111291

**Published:** 2024-10-25

**Authors:** Xinyao Li, Hao Ling, Zengyang He, Zihui Yang, Tao Jiang, Peng Huang, Jianguo Zeng

**Affiliations:** 1 College of Horticulture, Hunan Agricultural University, Changsha 410128, China; xinyaolin@stu.hunau.edu.cn (X.L.); linghao1996@stu.hunau.edu.cn (H.L.); 152550hzy@stu.hunau.edu.cn (Z.H.); 2 Hunan Key Laboratory of Traditional Chinese Veterinary Medicine, Hunan Agricultural University, Changsha 410128, China; yangzihui_2021@hunau.edu.cn (Z.Y.); jiangtao@jxutcm.edu.cn (T.J.); 3 College of Veterinary, Hunan Agricultural University, Changsha 410128, China

**Keywords:** broilers, growth performance, edible grass, antioxidant, SCFAs, microbiota

## Abstract

The hybrid plant edible grass (*Rumex patientia* L. × *Rumex tianschanicus* A. LOS), a member of the Rumex genus, presents a novel food source with a protein content of approximately 30–40%. By incorporating non-traditional feed sources, such as edible grass leaf powder (EGLP), into broiler production, costs could be reduced. The experimental subjects of this study were Arbor Acres (AA) broilers. A total of 300 newly hatched broilers were randomly divided into to five groups, with each group consisting of six cages, housing 10 birds per cage. The control group was fed a basal diet composed of maize and soybean meal. In the experimental groups, varying percentages (3%, 6%, 9%, and 12%) of EGLP were substituted for the corn–soybean meal in the basal diet. In the diet, for days 1–21, the corn content was reduced from 1.90% to 8.20%, and the soybean meal content was lowered from 1.50% to 6.00%. Similarly, in the diet, for days 22–42, the corn content was decreased from 1.17% to 7.00%, while the soybean meal content was reduced by 1.50% to 6.00%. The experiment lasted 42 days and was divided into two phases: the brooding phase (days 1 to 21) and the finishing phase (days 22 to 42). The results show that substituting 3% and 6% EGLP for corn and soybean meal significantly increased the average daily gain (ADG) during the brooding period compared to the control group (*p* < 0.01). Additionally, the group with a 3% substitution rate exhibited a significant increase in the average daily feed intake (ADFI) during the brooding phase (*p* < 0.01). Throughout the 42-day experiment, substituting 3% and 6% of EGLP for maize and soybean meal did not significantly affect the overall growth performance of broilers (*p* > 0.05). However, a 6% supplementation notably reduced the feed conversion ratio (FCR) (*p* < 0.01). Both the 3% (EG3) EGLP and 6% (EG6) EGLP replacement meals significantly enhanced the antioxidant capacity of broiler chickens, as indicated by the increased levels of the total antioxidant capacity (T-AOC), superoxide dismutase (SOD), and glutathione peroxidase (GSH-Px) (*p* < 0.01). Additionally, broilers supplemented with 3–12% showed a marked increase in cecal short-chain fatty acids (SCFAs) compared to the control group (*p* < 0.01). The 3% EGLP replacement diet also significantly boosted the prevalence of *Lactobacillus* in the cecum (*p* < 0.01). Furthermore, after EGLP supplementation, there was a higher abundance of SCFA-metabolizing bacteria, particularly *Alistipes* and *Christensenellaceae_R-7*, compared to pre-treatment (*p* < 0.05). The prevalence of *Clostridium* was significantly greater in the groups receiving 9% and 12% EGLP (*p* < 0.05), while *Butyrivibrio* levels were notably higher after supplementation with 12% EGLP (*p* < 0.05).

## 1. Introduction

With the expected rise in global populations and incomes, particularly in developing nations, there is likely to be a sharp increase in the demand for premium foods, such as meat, eggs, and milk. If feed efficiency remains constant, the global feed supply in terms of dry matter is projected to increase by 21%, reaching 1.3 billion tons between 2010 and 2025 [1]. However, protein feed continues to be a critical limiting factor in feed formulation. At present, most high-quality plant protein sources come from byproducts of oilseed processing, such as soybean meal, cottonseed meal, and rapeseed meal [2]. In China, soybean imports play a significant role, accounting for over 60% of all grain imports in 2020 and 2021. During this period, soybean imports exceeded 100 million tons [3]. The heavy reliance on grains, oilseeds, and soybeans—crops primarily intended for human consumption—for animal feed production poses a direct threat to food security [4]. To address this challenge, the development of alternative protein feed sources has become a crucial strategy. Researchers are investigating various approaches to diversify feed resources, including plant-based proteins (such as algal protein) [5,6], microbial proteins (such as single-cell protein) [7], and animal-derived proteins (such as insect protein) [8].

Plant leaves are abundant in essential amino acids (such as lucerne), vitamins, minerals, and bioactive compounds (such as basil, oregano, and thyme), making them a valuable resource as feed [9]. Consequently, their potential as feed additives has been extensively studied. Ginindza et al. [10] compiled data regarding alfalfa in broilers. Exceeding 5% lucerne adversely affects broiler performance, and enzymes may serve as a viable alternative. Leaf meals are particularly noted for their bioactive compounds, which can enhance the antioxidant capacity of both livestock and poultry. Numerous studies have demonstrated that incorporating specific herbs or plant leaf powders into the diets of broiler chickens can significantly boost their antioxidant defenses and improve growth performance and overall health. For instance, Gong et al. [11] reported that adding 3% kudzu leaf meal to broiler diets significantly increased serum T-AOC and glutathione peroxidase (GSH-Px) activity, while reducing serum malondialdehyde (MDA) levels. This effect is likely attributed to the high isoflavone content in kudzu leaves. Additionally, the inclusion of 2% mulberry leaf powder has been shown to enhance superoxide dismutase (SOD) activity in weaned pigs [12]. Flavonoids derived from alfalfa leaves have also been found to increase the activities of SOD, catalase (CAT), T-AOC, and GSH-Px in the bloodstream of Arbor Acre female broilers, while concurrently lowering MDA levels [13].

Leaf meal typically raises the diet’s crude fiber level in addition to its protein amount. At the moment, consuming modest levels of fiber (3–5%) can enhance gut health by raising the quantity of beneficial bacteria and intestinal villi that contain volatile short-chain fatty acids [14]. The microorganisms in the chicken cecum play a crucial role in breaking down complex plant-derived compounds, such as cellulose and other intricate carbohydrates, which are difficult for poultry to digest. Through fermentation, cecal bacteria produce energy primarily in the form of short-chain fatty acids (SCFAs), which consist of a hydrocarbon chain and a carboxylic acid group [15]. These acids can serve as an energy source for the birds [16]. For example, the inclusion of inulin has been shown to improve intestinal health by fostering the growth of beneficial bacteria like *Lactobacillus* and *Bifidobacterium*, and by increasing the production of SCFAs such as butyric acid, acetic acid, and propionic acid. These fatty acids also possess antibacterial properties, making them effective in combating *Salmonella* infections in young chickens [17]. Therefore, SCFAs play a key role in promoting a diverse and balanced microbial population in the gut, which is essential for maintaining overall gut health.

Edible grass (*Rumex patientia* L. × *Rumex tianschanicus* A. LOS) is a perennial species within the genus *Polygonum* in the family *Polygonaceae*. Functional plant-derived feed additives, such as *Rumex nepalensis* [18] and *Rumex nervosus* [19], enhance antioxidant and immunological characteristics, as well as increasing intestinal villi height and surface area, hence improving the growth performance of broiler chickens. This study aims to evaluate the impact of edible grass on growth performance, major intestinal metabolites (short-chain fatty acids), microbial communities, and antioxidant capacity in broilers. Edible grass is known to contain bioactive compounds with potential antioxidant properties that may enhance tissue oxidative stability and promote overall poultry health. In practical applications, edible grass could serve as a substitute for conventional feed ingredients like soybean meal, fish meal, and alfalfa, contributing to more diverse feed formulations, reducing costs, and offering potential health benefits through its antioxidant activity. This study provides a scientific basis for the use of edible grass in the animal feed industry, highlighting its multifaceted benefits, including growth promotion, microbial modulation, and antioxidant enhancement.

## 2. Materials and Methods

### 2.1. Experimental Materials

Joyherb Biotechnology Co., Ltd., a commercialization firm based in Changsha, China, provided the EGLP used in this study. The EGLP samples were air-dried and then ground using a grinder (JIASOUND Co., Ltd., Jinhua, China). The ground material was passed through a 0.425 mm mesh screen to achieve a uniform particle size. The contents of dry matter (DM), crude protein (CP), ether extract (EE), crude fiber (CF), calcium (Ca), and phosphorus (P) in the powder (Table 1) were analyzed following the standard procedures outlined by the Association of Official Analytical Chemists (AOAC) [20]. The total flavonoid and total polyphenol (Table 1) contents of the edible grass and between the different treatment groups were determined using commercial kits (Nanjing Jiancheng Biological Technology Co., Ltd., Nanjing, China). The amino acid content (Table S1) of the edible grass was determined using an Eusing Hitachi L-8900 Amino Acid Analyser (Hitachi Co., Ltd., Tokyo, Japan) [21].

### 2.2. Animals and Treatment

A total of 300 newly hatched Arbor Acres broilers were randomly allotted into 5 equal treatments (60 birds each), while each group was subdivided into 6 replicates (cages) each of 10 chicks during the experimental period extended from 1 day to 42 days of age. The basal diet used in this study was a corn–soybean meal, which served as the control group. The corn–soybean meal was replaced with EGLP in the basal diet at varying percentages of 3%, 6%, 9%, and 12%. This replacement resulted in a reduction in corn and soybean meal in the diet. Specifically, in the 1–21-day diet, corn was reduced from 1.90% to 8.20%, and the soybean meal was reduced from 1.5% to 6%. In the 22–42-day diet, corn was reduced from 1.17% to 7.00%, and soybean meal was reduced from 1.50% to 6.00%.

The diets were prepared according to the “Chicken Feeding Standard” (NY/T 33-2004) of the People’s Republic of China, which is the agricultural industry standard of the country [22]. The trial lasted 42 days, adhering to the feeding guidelines for broilers, and was divided into two phases: the starter phase (days 1 to 21) and the growing phase (days 22 to 42). The composition and nutritional content of the experimental diets for both phases are detailed in Table 2 and Table 3.

The broilers were housed in three-tiered stacked cages. From day 1 to day 7, the temperature was maintained at 34 °C ± 1 °C. Following this period, the temperature was gradually reduced by 1 °C per day until reaching 25 °C ± 1 °C, which was then maintained until the end of the trial on day 42. The barn’s relative humidity was kept at 50%, with regular ventilation and continuous 24 h lighting. The broilers had ad libitum access to both feed and water. Immunization was carried out in strict accordance with the Arbor Acres broiler immunization management manual.

### 2.3. Growth Performance

Each broiler was weighed and recorded on an empty stomach at 07:00 on the 1st, 21st, and 42nd day of the experiment. The birds underwent 12 h of fasting before being weighed. The amount of feed consumed by each pen was recorded over the starter and finisher periods. Mortality was recorded daily. Any bird that died was weighed, and the weight was used to adjust the feed conversion ratio (FCR).

These measurements were used to calculate the following parameters: average daily gain (ADG), average daily feed intake (ADFI), and feed conversion ratio (FCR).

ADFI = total feed intake/(test days * total number of test chicken)

ADG = (final body weight − initial body weight)/number of test days

FCR = total feed intake/total body weight gain

### 2.4. Sample Collection

On the 21st and 42nd days of the trial, one chicken with a weight comparable to the average was selected from each replicate in both the CS, EG3, EG6, EG9, and EG12 groups. Blood samples were collected from the chickens’ wing veins. Following this, the chickens were euthanized by administering an intravenous injection of a lethal dose of barbiturate, which was subsequently followed by cervical dislocation. The blood samples were left undisturbed for 1 h, before being centrifuged at 4000× *g* per minute to obtain the plasma, which settled above. The plasma was then rapidly frozen using liquid nitrogen and transported to the laboratory, where it was stored at −80 °C until being ready for antioxidant testing. The contents of their intestines were obtained from the cecum within 5 min after euthanasia. The contents were promptly placed in cryopreservation tubes and rapidly frozen using liquid nitrogen. The samples were then transported to the laboratory and stored at −80 °C until DNA extraction and the analysis of volatile fatty acids in the cecal digesta cloud be performed.

### 2.5. Measurement of Antioxidants and Volatile Short-Chain Fatty Acids

The T-AOC, SOD, and GSH-Px in the blood were measured using the corresponding enzyme-linked immunosorbent assay (ELISA) kit (Nanjing Jiancheng Biological Technology Co., Ltd., Nanjing, China). The method described by Franklin et al. [23] was used to assess the concentration of volatile SCFAs in the cecal contents. Specifically, 1 g of cecal digesta was defrosted and mixed with 2 milliliters of distilled water, followed by vortexing to ensure thorough blending. The samples were then centrifuged at 4 °C at 12,000× *g* for 10 min. Following centrifugation, 1 milliliter of the supernatant was transferred to a 2 milliliter tube and combined with 0.2 milliliter of metaphosphoric acid. After filtration through a 0.22 µm membrane, the sample was prepared for analysis. An aliquot of 1 microliter from the supernatant was analyzed using a gas chromatograph (GC-2010plus, Shimadzu, Japanese) equipped with a flame ionization detector (FID). The oven temperature was maintained at 120 °C for 15 min, and nitrogen gas was used as the carrier gas at a flow rate of 1.8 milliliters per minute. The minimum detection limit for each short-chain fatty acid was 0.1 millimoles per liter. The SCFAs in the samples were quantified using an external standard method (standards were purchased from Aladdin), and the constructed standard curves are shown in Figure S1.

### 2.6. DNA Extraction and PCR Amplification

The complete genomic DNA from microorganisms in the cecal contents was extracted using the E.Z.N.A. Soil DNA Kit (Omega Biotek, Norcross, GA, United States), in accordance with the manufacturer’s instructions [24]. The concentration of the extracted total microbial genomic DNA was determined using a NanoDrop^®^ ND-2000 (Thermo Scientific Inc., Waltham, MA, USA) spectrophotometer. Subsequently, 1.0% agarose gel electrophoresis was performed to confirm the integrity and purity of DNA. Upon the completion of the detection process, the DNA samples were stored at −80 °C to ensure stability until they were utilized in subsequent experiments. The V3-V4 hypervariable regions of the bacterial 16S rRNA gene were amplified using the ABI GeneAmp^®^ 9700 PCR thermal cycler. The primer pair used in this study consisted of the forward primer 338F (5′-ACTCCTACGGGAGGCAGCAG-3′) and the reverse primer 806R (5′-GGACTACHVGGGTWTCTAAT-3′). The PCR reaction mixture included the following components: 4 μL of 5 × Fast Pfu Buffer, 2 μL of 2.5 mM dNTPs, 0.8 μL of each primer (at a concentration of 5 μM), 0.4 μL of Fast Pfu Polymerase, and 10 ng of template DNA. The final volume of the reaction mixture was adjusted to 20 μL with ddH_2_O. The PCR amplification comprised four steps: An initial denaturation step at 95 °C for 3 min to ensure the complete denaturation of the template DNA, followed by 27 cycles. Each cycle consisted of a denaturation step at 95 °C for 30 s, an annealing step at 55 °C for 30 s, and an extension step at 72 °C for 45 s. After completing all cycles, a final extension step was performed at 72 °C for 10 min to ensure the complete synthesis of all PCR products. The PCR reaction was then terminated at 4 °C to maintain the stability of the PCR products. All samples were amplified in triplicate.

### 2.7. Sequencing Analysis and Bacterial Data Processing

PCR products were separated using 2% agarose gel electrophoresis and purified with the AxyPrep DNA Gel Extraction Kit (Axygen Biosciences, Union City, CA, USA), according to the manufacturer’s instructions. The purified PCR products were quantitatively analyzed using a Quantus™ Fluorometer (Promega, Madison, WA, USA). The purified amplicons were pooled in equimolar amounts to ensure the representation of each sample during sequencing. Paired-end sequencing was performed on the Illumina MiSeq PE 300 platform and NovaSeq PE 250 platform (Illumina, San Diego, CA, USA). The high-quality sequencing analysis of the pooled amplicon samples was conducted following the standard protocol provided by Majorbio Bio-Pharm Technology Co., Ltd. (Shanghai, China).The raw sequencing reads were deposited in the NCBI Sequence Read Archive (SRA) under project number PRJNA1133901. After demultiplexing, the sequences were quality-filtered using the fastp software (version 0.19.6) to ensure data integrity. The filtered sequences were then merged using the FLASH software (version 1.2.11) [18]. The methods for sequence processing and merging were based on relevant reviews by Chen et al. [25] and Magoc and Salzberg [26]. High-quality sequences were denoised using the DADA2 plugin in Qiime2 software (version 2020.2). The denoised sequences were classified as amplicon sequence variants (ASVs). The denoising method and its application followed research reports by Calamari et al. [27] and Bolyen et al. [28].

### 2.8. Statistical Analysis

Data on antioxidant levels and SCFAs were organized using Microsoft Excel 2016 and analyzed with IBM SPSS Statistics 25. A one-way analysis of variance (ANOVA), followed by Duncan’s multiple range test, was conducted to compare the groups. Additionally, linear and quadratic regression analyses were performed to explore the relationship between forage grass powder supplementation and various variables. The results are presented as means and standard deviations, with statistical significance set at *p* < 0.05 and high significance at *p* < 0.01. Production performance data were visualized using the Prism 10 software (Version 10.2.3), which applied a one-way ANOVA, including nonparametric or mixed methods, to examine group differences. The gut microbiota analysis was carried out using the Majorbio Cloud platform (https://cloud.majorbio.com), accessed on 8 July 2024. Where amplicon sequence variant (ASV) data were utilized to compute rarefaction curves and α-diversity indices, including Sobs, ACE, Shannon, and Simpson indices, calculated with Mothur version 1.30.1 [29]. A principal coordinate analysis (PCoA) based on Bray–Curtis dissimilarity was performed using the Vegan software package, version 2.5-3, to assess microbial community similarity. The Kruskal–Wallis H test evaluated differences in cecal microbiota, with significance set at *p* < 0.05. The Bonferroni correction (post hoc Tukey–Kramer) was applied to enhance analysis rigor, acknowledging that *p*-values above this threshold still indicated potential relationships. Bacterial taxa enrichment was identified using the Linear Discriminant Analysis (LDA) Effect Size (LEfSe) tool(Version 1.0) (http://huttenhower.sph.harvard.edu/galaxy/LEfSe, accessed on 8 July 2024.), following the methodology outlined by Segata et al. [30], with an LDA score > 4 used as the screening criterion. The correlation between gut microbiota and SCFAs and antioxidant indicators was analyzed using the RStudio software (Version 2023.06.2) and is represented as a Spearman correlation heatmap.

## 3. Results

### 3.1. Growth Performance of Chickens Fed Different Diets

**Figure 1:**
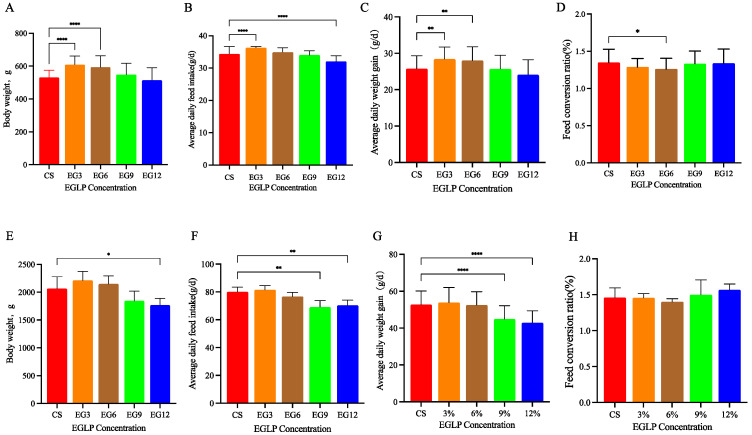
Broiler growth performance on corn–soybean (CS) and EGLP diets (3%~12%). (**A**) Body weight (BW) of 21-day broilers; (**B**) average daily feed intake (ADFI) of 21-day broilers; (**C**) average daily weight (ADG) of 21-day broilers; (**D**) feed conversion ratio; (**E**) body weight (BW) of 42-day broilers; (**F**) average daily feed intake (ADFI) of 42-day broilers; (**G**) average daily weight (ADG) of 42-day broilers; (**H**) feed conversion ratio. (*) 0.01 < *p* < 0.05; (**) *p* < 0.01; (****) *p* < 0.0001.

As illustrated in Figure 1A,B, replacing corn and soybean meal in the diet with 3% and 6% edible grass leaf powder (EGLP) significantly enhanced the body weight (BW) and average daily gain (ADG) of broilers from 1 to 21 days of age compared to the corn–soybean meal group (*p* < 0.01). Additionally, substituting the corn–soybean meal with 3% EGLP resulted in a substantial increase in the ADFI of broiler chickens aged 1 to 21 days (*p* < 0.01), as shown in Figure 1C. Furthermore, this dietary modification led to a significant reduction in the feed conversion ratio (FCR) from 1 to 21 days of age (*p* < 0.01) (Figure 1D). However, the inclusion of 3% and 6% EGLP in the diets did not affect the ADG and ADFI of broilers from 1 to 42 days of age compared to the corn–soybean meal group (*p >* 0.05) (Figure 1F). Supplementing broilers with 9% and 12% EGLP significantly decreased their daily growth and feed intake (*p* < 0.01) (Figure 1G), while the feed conversion ratio (FCR) of broiler chickens did not improve with 9% and 12% EGLP (*p* > 0.05) (Figure 1H). However, it did trend upward.

### 3.2. Antioxidant Properties of Chickens Fed Different Diets

Table 4 presents the results for 21-day-old broilers. The findings indicate that the EG3 and EG9 groups exhibited a significant increase in the total antioxidant capacity (T-AOC) and superoxide dismutase (SOD) levels (*p* < 0.01). However, the inclusion of EGLP did not result in any significant changes in the blood levels of GSH-Px (*p* > 0.05).

Table 5 displays the results for 42-day-old broilers. The findings demonstrate that including EGLP in the diet led to a significant elevation in both T-AOC and GSH-Px levels in broilers (*p* < 0.01).

### 3.3. Short-Chain Fatty Acids (SCFAs) of Chickens Fed Different Diets

Table 6 presents the results for 21-day-old broilers. The findings indicate that the addition of edible grass leaf powder (EGLP) had no significant effect on the acetic acid content in the cecum of these broilers (*p* > 0.05). However, the acetic acid levels exhibited a linear increase with the rising inclusion of EGLP. The isobutyric acid content in the EG6 to EG12 groups was significantly higher than that in the other groups (*p* < 0.05). Conversely, the butyric acid content in the EG3 group was significantly lower compared to that of the other groups (*p* < 0.05). Additionally, the isovaleric acid and valeric acid contents in the EG12 group were significantly elevated compared to those of the other groups (*p* < 0.05). Furthermore, the total SCFA content in the EG9 and EG12 groups was significantly greater than that in the other groups (*p* < 0.05).

Table 7 presents the results for 42-day-old broilers. As the EGLP levels in the diet increased, the butyric acid content in the cecum of the 42-day-old broilers initially decreased and then increased (*p* < 0.05). Notably, the EG12 group exhibited a rise in both the butyric acid and valeric acid contents in the cecum (*p* < 0.05). The EG3, EG9, and EG12 groups showed a significant increase in valeric acid compared to the control (CS) group (*p* < 0.05). However, the administration of EGLP did not lead to any significant changes in the levels of acetate, propionate, isobutyrate, and total SCFAs in the 42-day-old broilers (*p* > 0.05).

### 3.4. Effect of EGLP on the Intestinal Microbiota of Broilers

#### 3.4.1. Species Annotation and Assessment in Cecal Microbiota

The impact of leafy grass meal on the microbial community in the cecum of broiler chickens was investigated through the analysis of amplified products of the 16S rRNA gene. The results from the analyses conducted on days 21 and 42 indicate that, as the sequencing depth increased, the rarefaction curves for both the Shannon index and Sobs index flattened. This observation suggests that the quality of our sequencing data was satisfactory (Figure S2).

**Figure 2:**
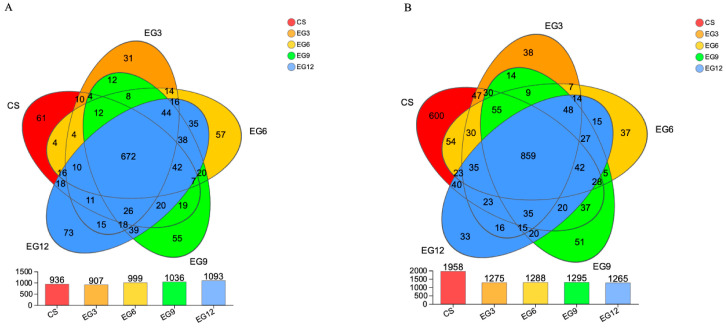
Venn diagrams of cecal microbiota at day 21 (**A**) and at day 42 (**B**).

The Illumina Miseq sequencing technique was employed to analyze the 338 F-806 R region of the bacterial 16S rRNA gene. This analysis yielded 1,605,249 high-quality sequences after 21 days and 1,529,923 high-quality sequences after 42 days. A total of 1141 operational taxonomic units (OTUs) was identified after 21 days, whereas 2307 OTUs were detected after 42 days, based on a sequence threshold of 97%. Figure 2A presents the Venn diagram illustrating the results from the 21-day analysis. We detected a total of 672 OTUs that were present in all samples. The CS group exhibited a total of 936 OTUs, of which 61 were unique. The GE3 group contained 907 OTUs, with 31 of them being unique. Similarly, the GE6 group comprised 999 OTUs, of which 57 were unique. The GE9 group included 1036 OTUs, with 55 of them being unique. Figure 2B displays the Venn diagram corresponding to day 42. A total of 859 OTUs was identified across all samples. The CS group exhibited a total of 1958 OTUs, of which 600 were unique. The EG3 group contained 1275 OTUs, with only 38 being unique. Similarly, the EG6 group had a total of 1288 OTUs, with 37 being unique OTUs. The EG9 group consisted of 1295 OTUs, of which 51 were unique. Finally, the EG12 group comprised 1265 OTUs, with 33 being unique.

**Figure 3:**
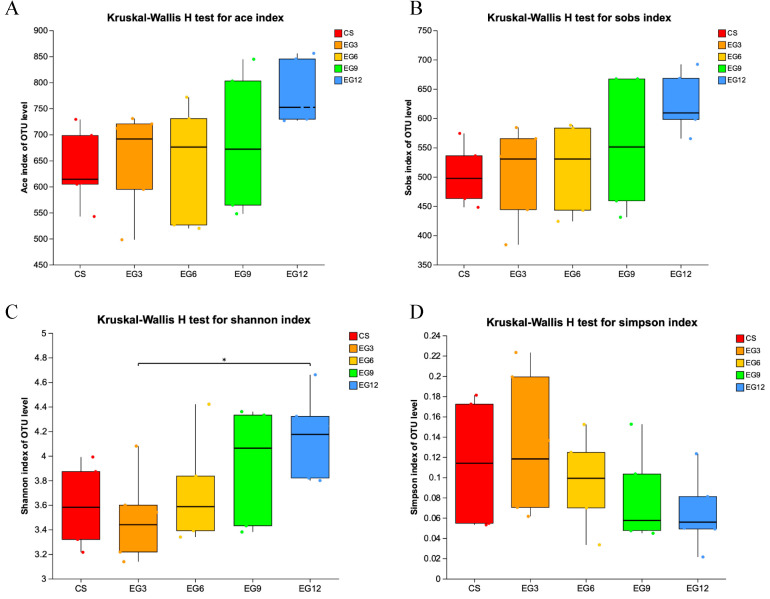
Analysis of the alpha diversity in the cecum of 21-day-old broilers. (**A**), Ace index of OUT level; (**B**), Sobs index of OUT level; (**C**), Shannon index of OUT level; (**D**), Simpson index of OUT level. (*) 0.01 < *p* < 0.05.

**Figure 4:**
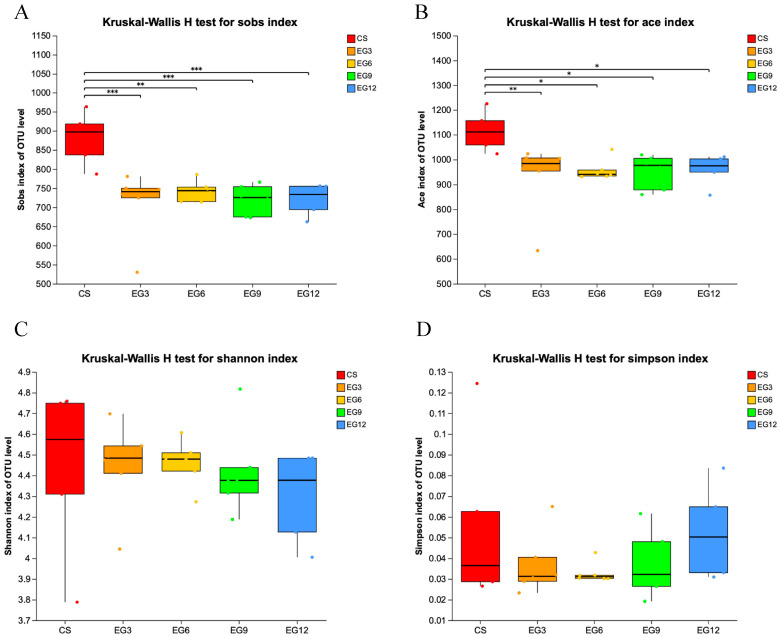
Analysis of the alpha diversity in the cecum of 42-day-old broilers. (**A**), Ace index of OUT level; (**B**), Sobs index of OUT level; (**C**), Shannon index of OUT level; (**D**), Simpson index of OUT level. (*) 0.01 < *p* < 0.05; (**) *p* < 0.01; (***) *p* < 0.001.

The alpha diversity analysis, which determines the microbial diversity in the cecum of 21-day-old broiler chickens, as measured by the Shannon and Sobs indices, was significantly higher in the EG12 group compared to the CS group (*p* < 0.05). However, the microbial diversity in the cecum of 42-day-old broiler chickens yielded contrasting results. Specifically, the cecal microbial diversity, as measured by the ace and Sobs indices, was significantly greater in the CS group than in the EG group (*p* < 0.05) (Figure 3 and Figure 4, and Table S2).

**Figure 5:**
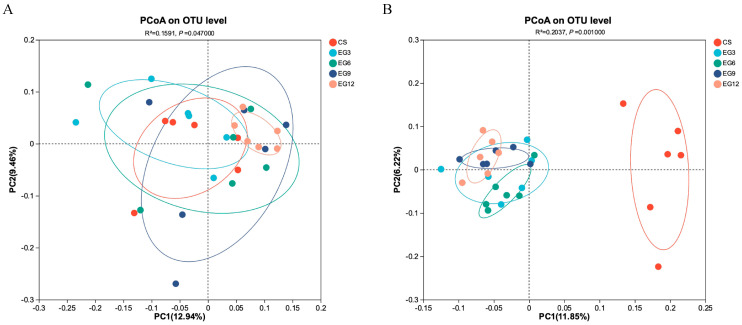
Effect of leaf grass feeding on the β-diversity analysis of microbiota at day 21 (**A**) and day 42 (**B**). *p* > 0.05 indicates no significant difference and *p* < 0.05 indicates significance.

An analysis of diversity was conducted to investigate the variations in the composition of the cecal microbial community across different groups, specifically the 3% addition group (EG3), 6% addition group (EG6), 9% addition group (EG9), 12% addition group (EG12), and the maize–soybean meal group (CS). A principal coordinate analysis (PCoA) was performed to examine the bacterial communities across the various treatments at two time points: 21 days and 42 days. The results of the PERMANOVA analysis indicated significant differences between the bacterial communities, with R^2^ values of 0.1591 (*p* = 0.0470) for Figure 5A and 0.2037 (*p* = 0.0010) for Figure 5B. These findings clearly demonstrate a distinct separation between the bacterial communities of the supplemented treatments (EG3, EG6, EG9, and EG12) and that of CS, particularly within the cecal bacterial colonies during the later stages of the study.

#### 3.4.2. Effects of EGLP on the Cecal Microbiota at the Phylum Level

**Figure 6:**
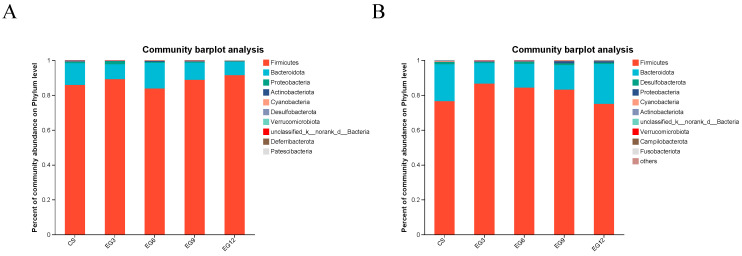
Effect of edible grass on the cecum microbiota at the phylum level at 21 days (**A**) and 42 days (**B**).

At 21 days of age, a grand total of 10 phyla were identified. The most prevalent phyla (constituting more than 1%) found in the cecal contents of both the control and EGLP broilers at 21 days old were mostly F_*Firmicutes*, F_*Bacteroidota*, and F_*Proteobacteria* (Figure 6A, Table S3). There was no statistically significant variation in the species composition between the 21-day-old broilers in the CS group and EGLP groups at the phylum level (*p* > 0.05).

At 42 days of age, a comprehensive count revealed the presence of 14 distinct phyla. Species with a relative abundance greater than 1% were chosen by arranging them in descending order based on their abundance. On the 42nd day, the major bacteria were F_*Firmicutes*, F_*Bacteroidota,* F_*Proteobacteria,* and F_*Desulfobacterota* (Figure 6B, Table S4). There was no significant difference in species between the CS and EGLP groups of 42-day-old broiler chickens at the phylum level (*p* > 0.05).

#### 3.4.3. Effects of EGLP on the Cecal Microbiota at the Genus Level

**Figure 7:**
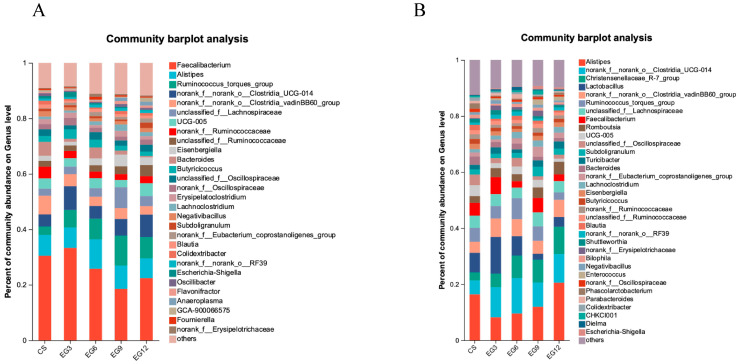
Effect of EGLP on the cecum microbiota at the genus level at 21 days (**A**) and 42 days (**B**).

At the genus level, a total of 157 genera was identified in the cecal contents of broiler chickens at 21 days of age, while 225 genera were identified at 42 days of age. At 21 days of age, 29 genera exhibited a relative abundance greater than 1% in the cecum of broiler chickens (Figure 7A). By 42 days of age, the number of genera with a relative abundance exceeding 1% in the cecum increased to 35 (Figure 7B).

**Figure 8:**
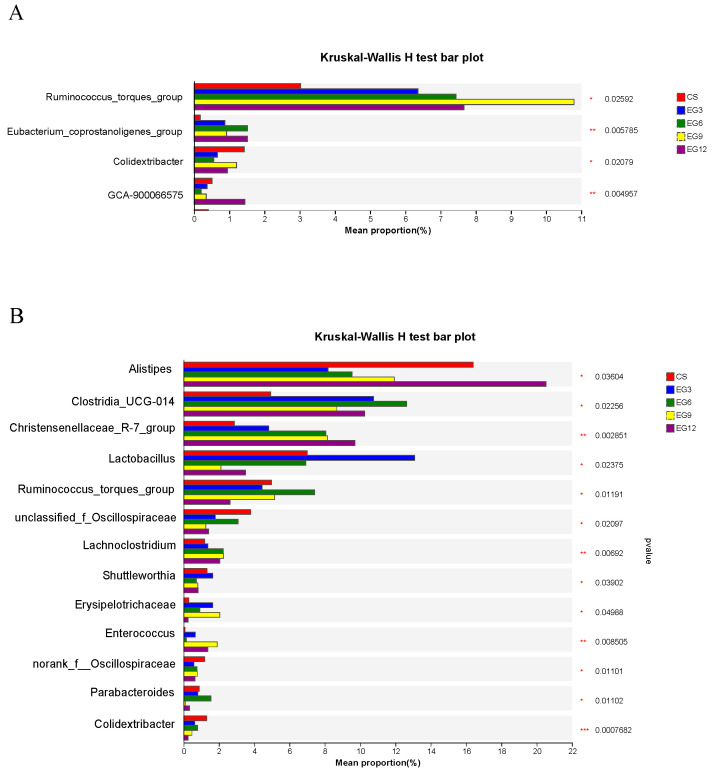
Relative abundances of genera that showed significant differences among samples from the cecum. A Kruskal–Wallis H test was used to evaluate the significance of differences between the indicated groups. (**A**), representative 21-day broiler genus-level differential microorganisms; (**B**), representative 42-day broiler genus-level differential microorganisms. * *p* < 0.05; ** *p* < 0.01; *** *p* < 0.001.

Out of them, four genera showed significant differences (*p* < 0.05) (Figure 8A, Table S5). In 21-day-old broilers, the EG group had a significantly greater relative abundance of g_*Ruminococcus_torques_group* (*p* = 0.024, *p*-adjust = 0.3215) and g_*Eubacterium_coprostanoligenes_group* (*p* = 0.006, *p*-adjust = 0.3027) compared to the CS group. The relative abundance of g_*Colidextribacter* (*p* = 0.021, *p*-adjust = 0.3264) in the EG3 and EG6 groups was considerably lower than that in the CS and EG12 groups. The prevalence of g_*GCA-900066575* (*p* = 0.005, *p*-adjust = 0.3027) in the EG12 group was substantially greater than that in the other groups.

Among the genera of broilers analyzedat 42-days-old, 12 genera exhibited significant differences (*p* < 0.05), as detailed in Figure 8B and Table S6. Notably, the abundance of g_*Lactobacillus* in the EG3 group was significantly higher (*p* = 0.024, *p*-adjust = 0.1781) than that in the other groups. The EG6 group showed similarities to the CS group, whereas the EG9 and EG12 groups displayed a marked reduction in abundance.

The prevalence of g_*Shuttleworthia* was significantly greater in both the CS and EG3 groups compared to the EG6, EG9, and EG12 groups (*p* = 0.036, *p*-adjust = 0.2148). Conversely, the prevalence of g_*Alistipes* (*p* = 0.039, *p*-adjust = 0.2195) was considerably lower in the EG3 and EG6 groups relative to the other groups.

Furthermore, the EG group exhibited a significantly greater relative abundance of g_*Christensenellaceae_R-7_group* (*p* = 0.003, *p*-adjust = 0.1399), g_*Clostridia* (*p* = 0.023, *p*-adjust = 0.1750), and g_*Lachnoclostridium* (*p* = 0.036, *p*-adjust = 0.1291) compared to the CS group. Additionally, the prevalence of g_*Ruminococcus_torques_group* (*p* = 0.011, *p*-adjust = 0.2148) in the EG6 group was significantly higher than that in the other groups. The prevalence of g_*Oscillospiraceae* (*p* = 0.021, *p*-adjust = 0.1685) in the EG9 and EG12 groups was notably decreased compared to the CS, EG3, and EG6 groups. 

#### 3.4.4. Effects of EGLP on Pathogenic Microorganisms and Butyrate-Producing Bacteria in Cecum of Broiler Chickens

**Figure 9:**
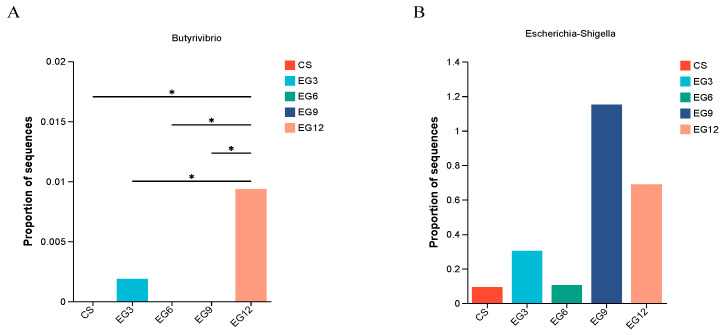
Effects of the leafy grass meal on pathogenic microorganisms and butyrate-producing bacteria in the cecum of broiler chickens. (**A**), Relative abundance of Butyrivibrio; (**B**), Relative abundance of Escherichia-Shigella. (*) 0.01 < *p* < 0.05.

Figure 9 presents the results for the broilers. In the EG12 group, the prevalence of g_*Butyrivibrio* in the cecum of 21-day-old broilers showed a significant increase (*p* = 0.001, *p*-adjust = 0.1691). In contrast, there were no significant variations in the proportion of g_*Escherichia-Shigella* in the cecum of 42-day-old broilers in the EG9 and EG12 groups compared to the CS, EG3, and EG6 groups. However, a slight upward trend was observed (*p* = 0.058, *p*-adjust = 0.2552).

#### 3.4.5. LEfSe Analysis of Chickens Fed Different Diets

**Figure 10:**
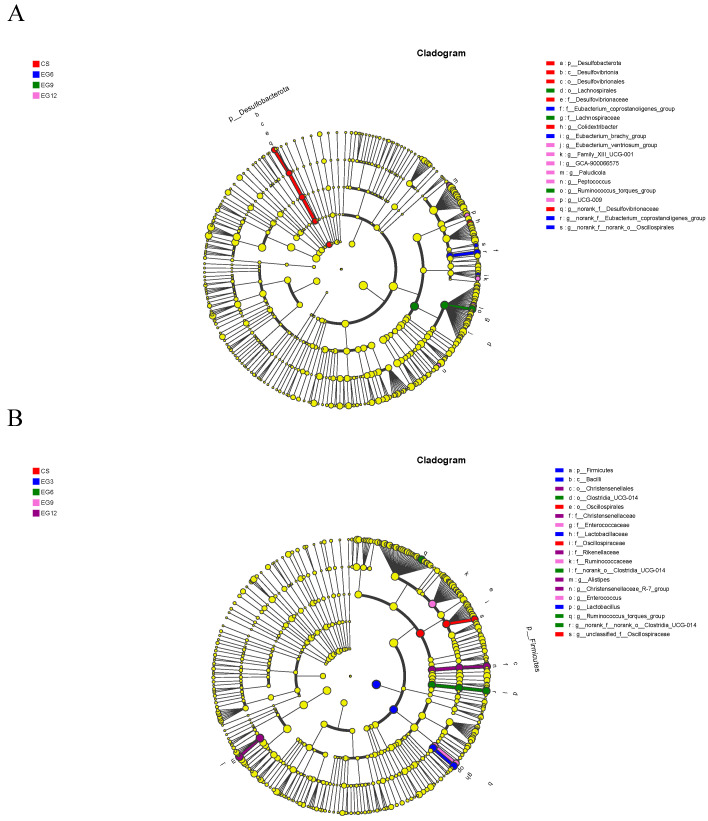
The LEfSE analysis was conducted to examine the multi-level species tree of cecal microbes in broilers at 21 (**A**) and 42 (**B**) days of age.

The LEfSe analysis approach was employed to identify the specific bacteria responsible for the observed statistical variations in the microbial communities among the three groups. This method was utilized to identify biomarkers. Based on the predetermined biomarker screening criteria (LDA score > 4), a total of 19 suitable species were identified at both 21 and 42 days of age. This information is presented in Figure 10 and Figure S3. Figure 10 illustrates the species that exhibited significant differences at the genus level in each group. In the CS group, the identified species were *Desulfovibrionaceae* (21 days old) and *Oscillospiraceae* (42 days old). In the EG3 group, the notable species was *Lactobacillus* (42 days old). In the EG6 group, the species were *Eubacterium_coprostanoligenes* and *Oscillospirales* (21 days old), and *Ruminococcus_toeques* and *Clostridia* (42 days old). The EG9 group showed significant findings for *Ruminococcus_toeques* (21 days old) and *Enterococcus* (42 days old). Finally, in the EG12 group, the species identified were *Eubacterium_ventriosum*, *Family_XIII_UCG-001*, *GCA-900066575*, *Paludicotahe*, and *Peptococcus* (21 days old), along with *Christensenellaceae-R-7* (42 days old).

#### 3.4.6. Correlations between SCFAs, Antioxidant Properties, and Cecal Microbiota

**Figure 11:**
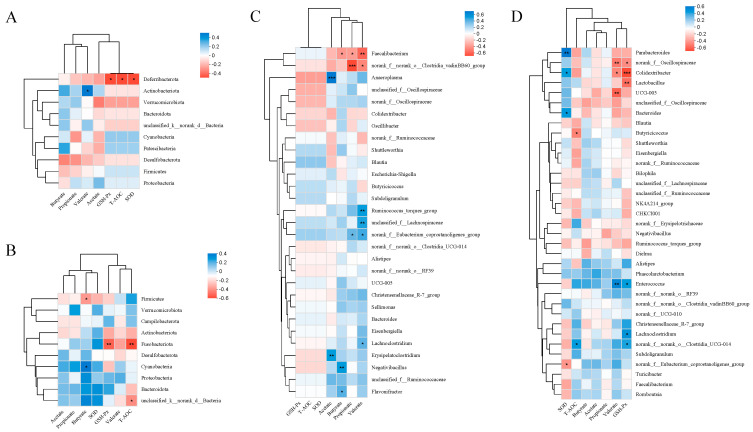
Heatmap of the Spearman correlations between SCFAs, antioxidant properties, and the cecal microbiota: 21-day phylum level (**A**) and genus level (**C**); 42-day phylum level (**B**) and genus level (**D**). (*) 0.01 < *p* < 0.05; (**) *p* < 0.01; (***) *p* < 0.001.

Figure 11A,B present the results of the correlation of cecum phylum-level microorganisms with antioxidant indicators and SCFAs for 21- and 42-day-old broilers. A significantly negative association was identified between the antioxidant properties (SOD, T-AOC, and GSH-Px) and *Deferribacteriota* in 21-day-old broilers. A significantly negative connection was identified between the antioxidant properties (SOD and T-AOC) and *Fusobacteriota* in 42-day-old broilers. Valerate had a significantly positive correlation with *Actinobacteriota* in broilers at 21 days of age. Butyrate had a significantly negative correlation with *Cyanobacteria* and Firmicutes at 42 days of life.

Figure 11C,D present the results of the correlation of cecum genus-level microorganisms with antioxidant indicators and SCFAs for 21- and 42-day-old broilers.

The cecal microorganisms of 21-day-old broilers exhibited no significant connection with antioxidant property indices at the genus level. Acetate exhibited a highly significant positive correlation with *Erysipelatoclostridium* and *Anaeroplasma* in 21-day-old broilers; butyrate demonstrated a significant positive correlation with *Flavonifractor*, while *Negativicacillus* displayed a significant negative correlation with *Faecalibacterium*. Propionate revealed a significant negative correlation with *Faecalibacterium* and a highly significant negative correlation with *Clostridia_vadinBB60*. Valerate indicated a significant negative correlation with *Clostridia_vadinBB60* and a highly significant negative correlation with *Faecalibacterium*.

SOD, an indicator of antioxidant quality in broilers at 42 days of age, exhibited a highly significant positive correlation with *Parabacteroides*, a significant positive correlation with *Solidertribacter* and *Bacteroides*, and a significant negative correlation with *Eubacterium_coprostanoligenes.* T-AOC, a measure of antioxidant capability, had a substantial positive connection with *Clostridia_UCG-014* and a significant negative correlation with *Butyricicoccus.* The antioxidant indicator GSH-Px exhibited a strong positive association with *Clostridia_UCG-014*, *Lachnoclostridium*, and *Enterococcus*; a highly significant negative correlation with *Lactobacillus* and *Colidextribacter*; and a substantial negative correlation with *Oscillospiraceae*, and an inverse link with *Oscillospiraceae*. Valeric acid had a highly significant negative correlation with *UCG-005* and Oscillospiraceae, as well as a strong negative correlation with *Colidextribacter*.

## 4. Discussion

### 4.1. Impact of Leaf Meal on the Growth Performance of Broilers

Feed ingredients can significantly influence gut bacterial communities and metabolite synthesis due to variations in particle size, type, and chemical composition. Plant leaf meal is extensively assessed for broiler nutrition, incorporating feed ingredients such as moringa, mulberry, cassava, and paper mulberry [31]. These leaf structures, including those of mulberry [32], purslane [33], Moringa [34], neem, and bamboo [35], are rich in bioactive substances and are commonly utilized as feed additives in animal nutrition, typically with a maximum inclusion rate of 1%. These bioactive compounds serve as alternatives to antibiotics, promoting animal health. Consequently, leaf resources are integrated into cattle and poultry feed both as essential ingredients and as functional additives.

Our research findings indicate that substituting the corn–soybean meal with EGLP at concentrations of 3% and 6% in broilers aged 21 and 42 days does not adversely affect body weight gain, ADFI, or FCR. In fact, a concentration of 3% EGLP has the potential to enhance growth performance. The results suggest that growth performance is optimized when soybean meal is replaced with 3% foliar grass meal. However, it remains uncertain whether substituting soybean meal with less than 3% foliar grass meal, such as replacing 0.75% soybean meal with 1.5% foliar grass meal or using a lower replacement ratio, would yield superior results. Further research is needed to investigate the effects of such substitutions. According to Wang et al. [33], incorporating 1% to 3% purslane into the standard diet of broiler chickens can improve their ADG and reduce the feed conversion ratio (FCR). Hence, substituting edible grass with a foliar content of less than 3% holds promise in stimulating the growth of broilers.

### 4.2. Leaf Meal Improves Antioxidant Properties in Broilers

In fact, these concentrations may even enhance growth performance. The bioactive compounds found in leaf meal are known to boost the antioxidant activity in livestock and poultry. Studies have demonstrated that incorporating leaf powder from certain plants into broiler diets can improve antioxidant capacity, growth performance, and overall health. For instance, the use of kudzu leaf powder resulted in a significant increase in the activities of serum T-AOC and GSH-Px, while simultaneously reducing the levels of serum MDA. This effect is likely attributed to the high concentration of isoflavones in kudzu leaves [11]. Research has indicated that flavonoids derived from alfalfa leaves can enhance the overall antioxidant capacity (T-AOC) and the effectiveness of antioxidant enzymes in the bloodstream. Additionally, these flavonoids have been found to lower levels of MDA [13]. Furthermore, a small amount of moringa leaf was shown to increase SOD levels in the blood and the expression of SOD1 mRNA [36]. The application of mistletoe leaf powder has also been reported to elevate the serum concentrations of glutathione peroxidase and catalase [37]. We hypothesize that bioactive compounds in leaf meal have a substantial impact on enhancing broiler performance, whether they are added in small quantities or used as a substitute for certain feed components. However, incorporating higher amounts of EGLP as a substitute for maize–soybean meal in basic diets may impede growth performance. This finding is consistent with those of previous research, which generally recommends a maximum inclusion rate of 10% leaf meal in broiler diets [38,39,40,41,42]. The suppression of growth observed at higher concentrations is likely attributable to the presence of anti-nutritional factors and the high fiber content found in the leaves. The summary by Tejeda et al. indicates that 1.5% wheat fiber, 1% inulin, 3% oat hulls, soyhulls, and 6% wood shavings enhanced feed efficiency (FE), but 10% oat hulls, 9% sunflower meal, and soyhulls led to a fall in efficiency [15]. Srivastava [43] determined that the excessive incorporation of mulberry leaves and paper strips adversely impacts cattle performance and health, hence becoming a significant factor in the utilization of mulberry leaf resources. Ding et al. [44] discovered that fermented mulberry leaves enhanced the performance of yellow-feathered broiler chicks relative to unfermented mulberry leaves and conventional soybean diets.

### 4.3. Impact of Leaf Meal on Intestinal Metabolites of Broilers

In terms of feed materials, the objective is to provide a balanced supply of energy from carbohydrates [45], fats [46], and proteins [47]. According to the literature, proteins, fats, bacteria, and antibiotics can significantly influence the composition of SCFAs in the intestines of broilers. SCFAs, which are byproducts of gut microbial activity, play a crucial role in the host’s energy metabolism. They nourish intestinal cells helping to maintain the integrity of the protective cell layer, regulate cell growth, support a balanced immune system, stimulate skeletal muscle formation, and contribute to the overall health and development of livestock and poultry [48].

Fiber in feed is widely recognized as a primary substrate for SCFAs, the main metabolites produced in the gut, and a key factor influencing their production. Leaf meals rich in protein, such as alfalfa and moringa, may serve as alternatives to soybean meal. These plant sources may be utilized in limited amounts for monogastric animals due to their elevated fiber content. Elevated concentrations of leaf meal augment the crude fiber content of the diet, potentially influencing animal growth performance. For instance, Tejeda and Kin [49] found that adding 12% and 19% soybean hulls (which contain 33% crude fiber) significantly decreased the final body weight of 21-day-old broilers while improving their feed conversion ratio. In contrast, adding 6% soybean hulls (which increased crude fiber by 2%) had no effect on growth. Our findings similarly demonstrate that EGLP elevated SCFA levels in the cecum; however, butyrate levels in 21-day-old EG3 broilers and 42-day-old EG3 and EG6 broilers were comparatively lower than those in other groups. This suggests that small amounts of EGLP may reduce butyrate concentration due to the presence of secondary metabolites, although further research is needed to confirm this.

The significant increase in SCFAs observed with higher dosages of EGLP can be largely attributed to its dietary fiber content. Walugembe et al. [50] reported that a higher dietary fiber intake decreases butyrate levels without affecting propionate and acetate concentrations. Additionally, SCFA levels did not enhance the growth performance of broilers fed a high-fiber diet. Therefore, it cannot be conclusively stated that increasing fiber alone enhances broiler growth performance by boosting SCFA production, as high-fiber leaf meals may introduce anti-nutritional factors and reduce the availability of carbohydrates. Russell et al. [51] found that individuals on a high-fiber diet had a significant increase in butyrate-producing bacteria, particularly *Roseburia* (including *Eubacterium rectale*), compared to those on low-carbohydrate, low-fiber diets. In our study, the abundance of Butyrivibrio, a key butyrate-producing genus, was significantly increased by the 12% EGLP, likely contributing to the higher butyrate levels in the EG12 group. LEfSE analysis at 21 days indicated that Desulfovibrionaceae was the predominant strain in the cecum of broilers fed a corn–soybean diet. The presence of both *Desulfovibrionaceae* and *Faecalibacterium* may enhance butyrate production, which could be beneficial in preventing disorders associated with low butyrate levels [52]. A reduction in soybean meal content could decrease the abundance of *Desulfovibrionaceae* abundance, potentially explaining the lower butyrate levels observed in the EG3 group. In summary, small amounts of EGLP lead to an overall increase in SCFA levels in the cecum, which may significantly contribute to the growth-promoting effects of low-concentration leaf meals.

### 4.4. Impact of Leaf Meal on the Intestinal Microbiota of Broilers

The gut microbiota plays a diverse and crucial role in maintaining host health by aiding digestion, regulating the immune system, defending against harmful bacteria, influencing the nervous system and behavior, and preserving gut barrier integrity [53]. Research indicates that incorporating lucerne meal into diets can decrease the diversity of microorganisms in the cecal microbiota [54], which aligns with our study’s results. Notably, *Lactobacillus*, recognized for its ability to enhance intestinal barrier integrity, strengthen the immune system, and reduce gut inflammation [55], showed a significant increase in abundance in our findings. Wang et al. [33] discovered that adding 1% to 3% purslane to broiler feed improved growth performance and reduced the feed conversion ratio(FCR), partly by increasing *Lactobacillus* abundance while decreasing *Escherichia-Shigella* levels. Similarly, Zheng et al. [56] reported that feeding hens with 5% and 8% lucerne meal resulted in a higher presence of *Lactobacillus* in the digestive system, particularly in the cecum. These observations align with our findings in the EG3 group, where Lactobacillus was the dominant bacterium at 42 days, as demonstrated by LEfSE analysis and the rank-sum test. In contrast, *Alistipes* and *Christensenellaceae_R-7* were the predominant bacteria in the EG12 group (Figure 8B). *Alistipes*, known for synthesizing acetate and propionate [57], likely served as a major source of SCFAs in the EGLP group. However, *Christensenellaceae_R-7*, which is negatively correlated with feed efficiency, acetate, and propionate concentrations, and is associated with lower body fat, increased proportionally with EGLP levels. This correlation may explain the reduced body weight observed in the EG9 and EG12 groups, where we also noted higher levels of the harmful bacteria Escherichia-Shigella.

## 5. Conclusions

Substituting corn–soybean feed with 3% to 6% EGLP (leaf meal) enhanced broilers’ growth performance. The growth-enhancing effects of low EGLP concentrations were primarily attributed to an elevated antioxidant capacity (T-AOC, SOD, and GSH-Px), improved intestinal fermentation resulting from increased dietary fiber concentration, and a rise in beneficial bacteria such as *Lactobacillus* spp. in the intestinal tract. A 12% inclusion of EGLP diminished the growth performance mostly due to elevated fiber content, increased concentrations of antinutrients, expansion of particular gut bacteria (e.g., *Christensenellaceae_R-7*), and proliferation of pathogenic bacteria.

## Figures and Tables

**Table 1:** Chemical composition of edible grass (on an air-dried basis).

Items	Edible Grass	Soybean Meal	Corn
DM, %	89.50	89.00	86.00
AME, MJ/kg	4.53	9.83	13.56
CP, %	29.97	44.00	8.70
CF, %	18.00	5.20	1.60
EE, %	3.00	1.90	3.60
Ash, %	11.62	6.10	1.40
Ca, %	0.70	0.33	0.02
P, %	0.39	0.62	0.27
Total flavonoids, mg/g	19.8	/	/
Total phenolic acid, mg/g	1.30	/	/

Note: DM CP stands for crude protein, CF stands for crude fiber, EE stands for ether extract, Ash stands for crude ash, Ca stands for calcium, and P stands for phosphorus. The data on soybean meal and corn were sourced from the “Chicken Feeding Standard” (NY/T 33-2004) of the People’s Republic of China.

**Table 2:** Ingredients and nutrient levels of the initial phase diets (on an air-dried basis).

Item	CS ^1^	EG3 ^2^	EG6 ^3^	EG9 ^4^	EG12 ^5^
Ingredients composition (%)					
Corn	54.96	53.06	50.96	48.86	46.76
Soybean meal	36.00	34.50	33.00	31.50	30.00
Soybean oil	4.60	5.00	5.60	6.20	6.80
Eidble grass	0.00	3.00	6.00	9.00	12.00
55% L-Lysine	0.56	0.56	0.56	0.56	0.56
98.5% DL-Methionine	0.22	0.22	0.22	0.22	0.22
CaHPO_4_	1.56	1.56	1.56	1.56	1.56
Limestone	1.20	1.20	1.20	1.20	1.20
60% Choline chloride	0.10	0.10	0.10	0.10	0.10
Sodium chloride	0.30	0.30	0.30	0.30	0.30
Premix ^6^	0.50	0.50	0.50	0.50	0.50
Total	100.00	100.00	100.00	100.00	100.00
Nutrient levels ^7^, % of DM					
ME,MJ/kg	12.55	12.55	12.55	12.55	12.50
Crude protein	20.00	20.00	20.00	20.00	20.00
Lysine	1.46	1.44	1.43	1.42	1.41
Methionine	0.55	0.53	0.52	0.52	0.52
Calcium	0.88	0.89	0.91	0.92	0.92
Available phosphorus	0.43	0.43	0.42	0.42	0.42
Crude fiber	2.70	3.40	3.80	4.20	4.60

^1^ CS: Corn–soybean meal diet (basic diet); ^2^ EG3: 3% EGLP-supplemented; ^3^ EG6: 6% EGLP-supplemented; ^4^ EG9: 9% EGLP-supplemented; ^5^ EG12: 12% EGLP-supplemented; ^6^ Provides per kilogram of diet: Vitamin A, 13,000 IU; Vitamin B1, 3.0 mg; Vitamin B2, 10.0 mg; Vitamin B6, 6 mg; Vitamin B12, 39 ug; Vitamin D3, 3800 IU; Vitamin E, 50 IU; Vitamin K3, 40 mg; Niacin, 4 mg; D-pantothenic acid, 16.0 mg; Folic acid, 2.5 mg; Biotin, 0.24 mg; Antioxidants, 50 mg; Cu, 9 mg; Fe, 100 mg; Zn, 110 mg; Mu, 129 mg; I, 1.8 mg; Se, 0.6 mg. ^7^ Nutrient levels were calculated values.

**Table 3:** Ingredients and nutrient levels of the second phase diets (on an air-dried basis).

Item	CS ^1^	EG3 ^2^	EG6 ^3^	EG9 ^4^	EG12 ^5^
Ingredients composition (%)					
Corn	60.78	59.61	57.78	55.88	53.78
Soybean meal	30.00	28.50	27.00	25.50	24.00
Soybean oil	5.20	5.20	5.20	5.60	6.20
Eidble grass	0.00	3.00	6.00	9.00	12.00
55% L-Lysine	0.30	0.30	0.30	0.30	0.30
98.5% DL-Methionine	0.15	0.15	0.15	0.15	0.15
CaHPO_4_	1.67	1.67	1.67	1.67	1.67
Limestone	1.00	1.00	1.00	1.00	1.00
60% Choline chloride	0.10	0.10	0.10	0.10	0.10
Sodium chloride	0.30	0.30	0.30	0.30	0.30
Premix ^6^	0.50	0.50	0.50	0.50	0.50
Total	100.00	100.00	100.00	100.00	100.00
Nutrient levels ^7^, % of DM					
ME,MJ/kg	13.00	13.00	13.00	13.00	13.00
Crude protein	18.00	18.00	18.00	18.10	18.20
Lysine	1.12	1.09	1.09	1.07	1.06
Methionine	0.43	0.43	0.43	0.43	0.43
Calcium	0.81	0.84	0.84	0.86	0.87
Available phosphorus	0.42	0.43	0.43	0.43	0.43
Crude fiber	2.80	3.20	3.60	4.10	4.50

^1^ CS: Corn–soybean meal diet (basic diet); ^2^ EG3: 3% EGLP-supplemented; ^3^ EG6: 6% EGLP-supplemented; ^4^ EG9: 9% EGLP-supplemented; ^5^ EG12: 12% EGLP-supplemented; ^6^ Provides per kilogram of diet: Vitamin A, 13,000 IU; Vitamin B1, 3.0 mg; Vitamin B2, 10.0 mg; Vitamin B6, 6 mg; Vitamin B12, 39 ug; Vitamin D3, 3800 IU; Vitamin E, 50 IU; Vitamin K3, 40 mg; Niacin, 4 mg; D-pantothenic acid, 16.0 mg; Folic acid, 2.5 mg; Biotin, 0.24 mg; Antioxidants, 50 mg; Cu, 9 mg; Fe, 100 mg; Zn, 110 mg; Mu, 129 mg; I, 1.8 mg; Se, 0.6 mg. ^7^ Nutrient levels were calculated values.

**Table 4:** Effect of EGLP on the antioxidant properties in the serum of broiler chicks that were 21 days old.

Item	T-AOC, mmol/L	SOD, U/mL	GSH-Px, U/mL
CS ^1^	0.74 ^c^	23.79 ^ab^	844.27
EG3 ^2^	0.98 ^ab^	33.59 ^a^	838.58
EG6 ^3^	1.17 ^a^	33.50 ^a^	973.28
EG9 ^4^	0.77 ^c^	29.06 ^a^	831.94
EG12 ^5^	0.83 ^bc^	15.10 ^b^	833.83
SEM	0.15	11.52	223.35
*p*-Value	0.006	0.034	0.807
Linear	0.926	0.122	0.934
Quadratic	0.005	0.004	0.665

^1^ CS: Corn–soybean meal diet (basic diet); ^2^ EG3: 3% EGLP-supplemented; ^3^ EG6: 6% EGLP-supplemented; ^4^ EG9: 9% EGLP-supplemented; ^5^ EG12: 12% EGLP-supplemented. Mean values of different superscripts (a,b,c) in the same column differed between CS and EG3, EG6, EG9 and EG12 (*p* < 0.05).

**Table 5:** Effect of EGLP on the antioxidant properties in the serum of broiler chicks that were 42 days old.

Item	T-AOC, mmol/L	SOD, U/mL	GSH-Px, U/mL
CS ^1^	0.74 ^c^	31.24 ^a^	956.92 ^b^
EG3 ^2^	1.02 ^ab^	31.56 ^a^	1126.96 ^ab^
EG6 ^3^	0.89 ^bc^	31.86 ^a^	1016.37 ^ab^
EG9 ^4^	1.11 ^a^	23.68 ^b^	1201.66 ^a^
EG12 ^5^	0.90 ^bc^	32.67 ^a^	1158.97 ^a^
SEM	0.03	11.52	30.42
*p*-Value	<0.001	0.004	0.049
Linear	0.016	0.111	0.020
Quadratic	0.005	0.056	0.215

^1^ CS: Corn–soybean meal diet (basic diet); ^2^ EG3: 3% EGLP-supplemented; ^3^ EG6: 6% EGLP-supplemented; ^4^ EG9: 9% EGLP-supplemented; ^5^ EG12: 12% EGLP-supplemented. Mean values of different superscripts (a,b,c) in the same column differed between CS and EG3, EG6, EG9 and EG12 (*p* < 0.05).

**Table 6:** Effect of EGLP on the SCFAs in the serum of broiler chicks that were 21 days old.

Item	Acetate ug/g	Propionate ug/g	Isobutyrate ug/g	Butyrate ug/g	Isovalerate ug/g	Valerate ug/g	Total SCFAs ug/g
CS ^1^	5156.35	801.71 ^b^	116.23 ^b^	1491.60 ^a^	104.60 ^b^	121.73 ^b^	7792.31 ^b^
EG3 ^2^	5937.51	836.97 ^b^	129.51 ^b^	1182.03 ^b^	116.02 ^b^	120.96 ^b^	8323.0 ^b^
EG6 ^3^	5841.06	883.42 ^b^	157.17 ^ab^	1712.61 ^a^	137.43 ^b^	147.25 ^b^	8878.93 ^b^
EG9 ^4^	7026.81	892.60 ^b^	136.84 ^b^	1542.31 ^a^	120.92 ^b^	150.37 ^b^	9868.86 ^ab^
EG12 ^5^	7513.49	1565.40 ^a^	215.40 ^a^	2105.40 ^a^	234.55 ^a^	223.97 ^a^	11,858.21 ^a^
SEM	327.60	69.53	10.41	116.76	13.29	9.02	454.12
*p*-Value	0.156	<0.001	0.023	0.089	0.012	<0.001	0.041
Linear	0.016	<0.001	0.04	0.032	0.003	<0.001	0.003
quadratic	0.794	0.008	0.287	0.214	0.089	0.030	0.334

^1^ CS: Corn–soybean meal diet (basic diet); ^2^ EG3: 3% EGLP-supplemented; ^3^ EG6: 6% EGLP-supplemented; ^4^ EG9: 9% EGLP-supplemented; ^5^ EG12: 12% EGLP-supplemented. Mean values of different superscripts (a,b) in the same column differed between CS and EG3, EG6, EG9 and EG12 (*p* < 0.05).

**Table 7:** Effect of EGLP on the SCFAs in the serum of broiler chicks that were 42 days old.

Item	Acetate ug/g	Propionate ug/g	Isobutyrate ug/g	Butyrate ug/g	Isovalerate ug/g	Valerate ug/g	Total SCFAs ug/g
CS ^1^	4581.33	1257.09	170.13	1174.64 ^ab^	231.01	140.93 ^b^	7555.15
EG3 ^2^	4626.31	1137.17	174.64	1020.04 ^c^	173.00	171.99 ^ab^	7303.15
EG6 ^3^	4415.21	1026.37	184.19	788.57 ^c^	172.06	157.55 ^b^	6743.95
EG9 ^4^	4913.53	1149.89	182.04	1113.10 ^ab^	177.74	192.58 ^ab^	7728.90
EG12 ^5^	6116.82	1305.28	213.28	1473.76 ^a^	209.52	226.85 ^a^	9545.52
SEM	275.72	53.80	7.61	71.23	8.76	177.98	405.11
*p*-Value	0.298	0.527	0.431	0.035	0.116	0.032	0.247
Linear	0.089	0.778	0.091	0.142	0.521	0.003	0.125
quadratic	0.567	0.093	0.516	0.006	0.012	0.247	0.096

^1^ CS: Corn–soybean meal diet (basic diet); ^2^ EG3: 3% EGLP-supplemented; ^3^ EG6: 6% EGLP-supplemented; ^4^ EG9: 9% EGLP-supplemented; ^5^ EG12: 12% EGLP-supplemented. Mean values of different superscripts (a,b,c) in the same column differed between CS and EG3, EG6, EG9 and EG12 (*p* < 0.05).

## Data Availability

The data presented in this study are available upon request from the corresponding author. The availability of the data is restricted to investigators based in academic institutions.

## Supplementary Materials

The following supporting information can be downloaded at: https://www.mdpi.com/article/10.3390/antiox13111291/s1, Figure S1: Standard curves for SCFAs. Figure S2: Dilution curves for sequencing of broiler cecum contents. Figure S3: LDA Discriminant Bar Chart of Cecal Microbiota in Broilers at 21 and 42 Days Old; Table S1: Amino acid Composition of Edible Grass (air-dried basis). Table S2: Microbial α-diversity in the cecum of 21- and 42-day-old broilers. Table S3: The composition and percentage of cecal microbiota at the phylum level in broilers at 21-days-old of age. Table S4: The composition and percentage of cecal microbiota at the phylum level in broilers at 42-days-old of age. Table S5: Tests for multiple differences in cecum microbial diversity in 21-day-old broilers. Table S6: Tests for multiple differences in cecum microbial diversity in 42-day-old broilers.
