# Supplementary material for: Effects of Edible Grass (Rumex patientia L. × Rumex tianschanicus A. LOS) Leaf Powder on Growth Performance, Antioxidant Properties, Cecal Short-Chain Fatty Acids, and Microbial Community Levels in Broilers
Source: Antioxidants (Basel). 2024 Oct 25;13(11):1291. doi: 10.3390/antiox13111291 (PMC11590973; doi:10.3390/antiox13111291)
Supplement: Supplementary file 1 [file antioxidants-13-01291-s001.zip › antioxidants-3219919-supplementary.pdf]

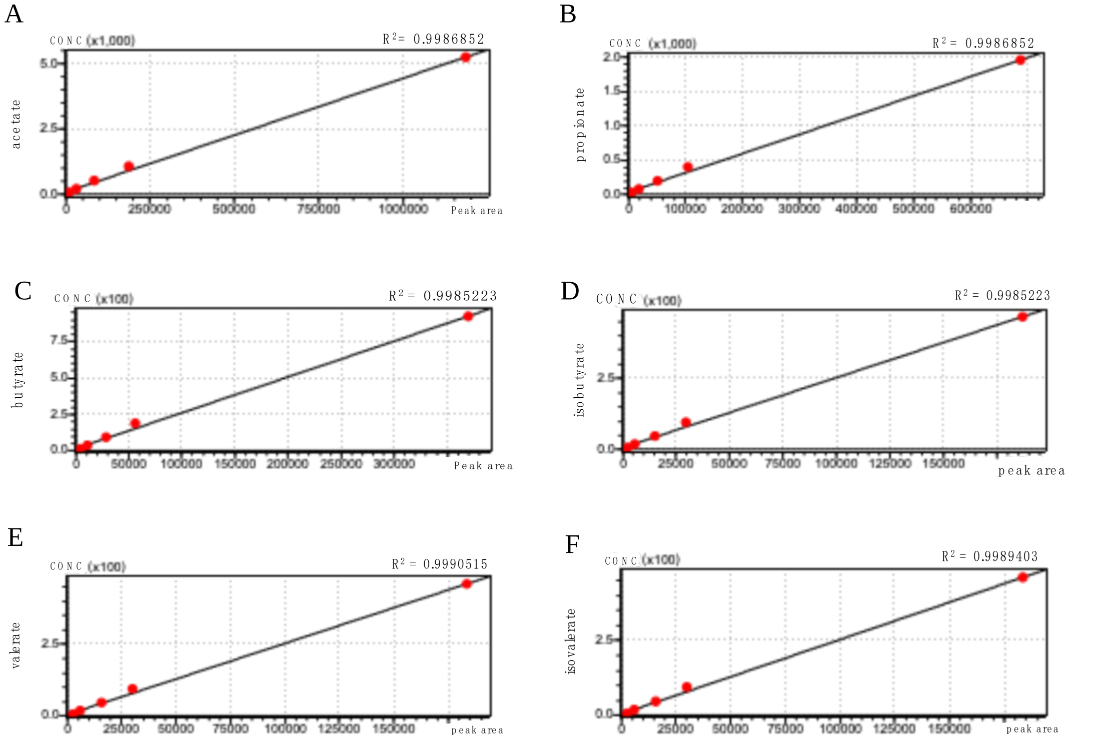


**Supplementary Figure S1**: Standard curves for SCFAs.(A,acetate; B, propionate; C, butyrate; D isobutyrate; E valerate; F isovalerate.)

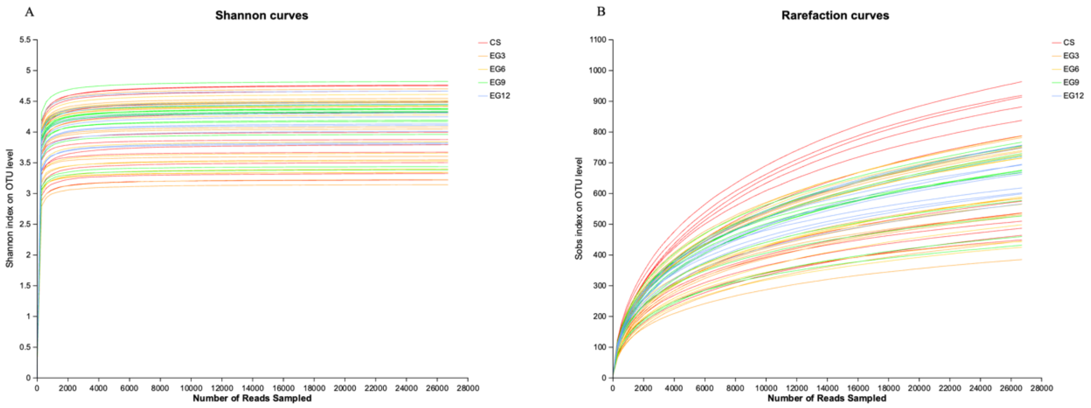


**Supplementary Figure S2:** Dilution curves for sequencing of broiler cecum contents (with Shannon and Sobs indices).

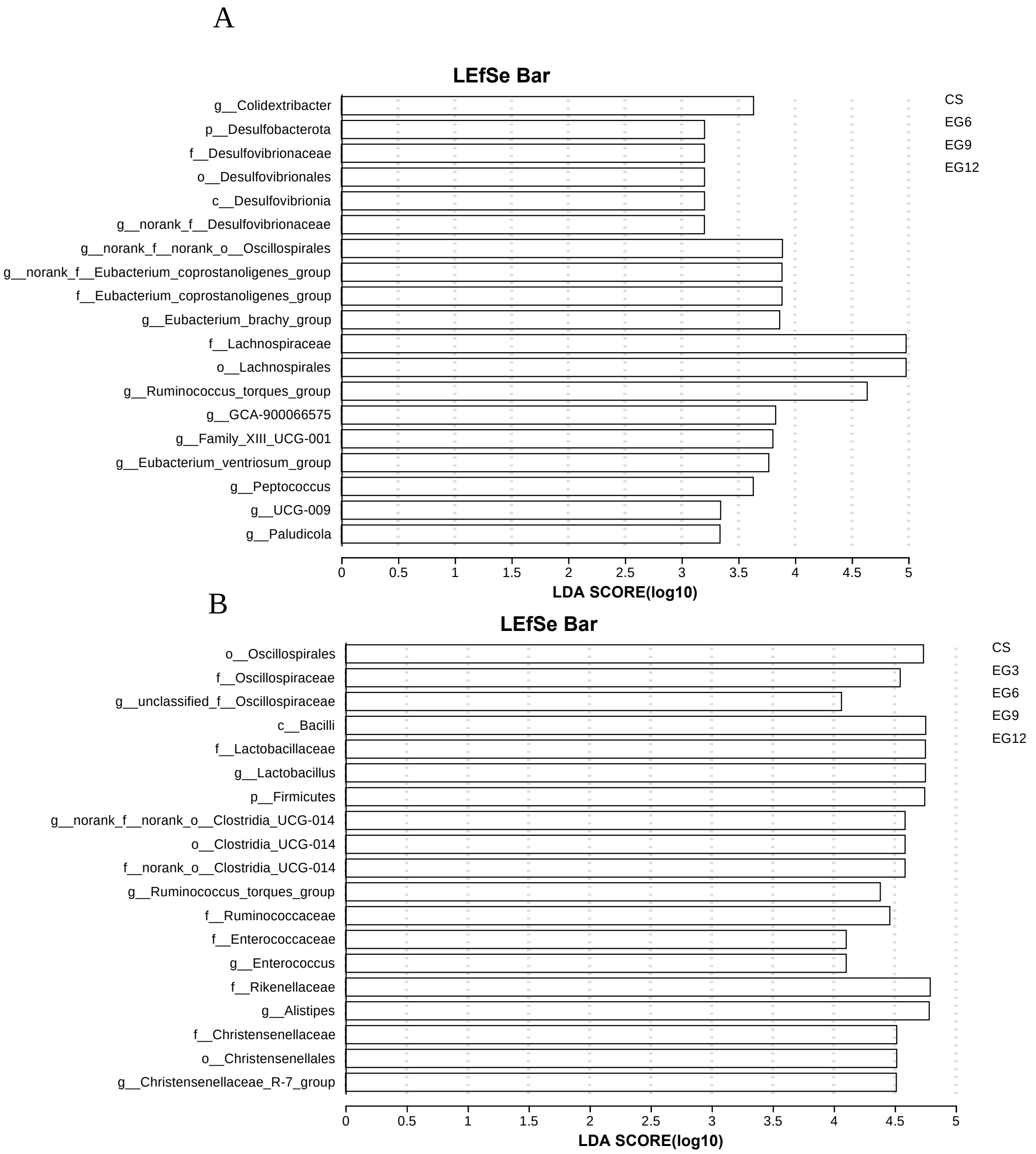


**Supplementary Figure S3**: LDA Discriminant Bar Chart of Cecal Microbiota in Broilers at 21 and 42 Days Old

**Supplementary Table S1:** Amino acid Composition of Edible Grass( air-dried basis).

| Items | Edible Grass | Soybean meal | Corn |
|---|---|---|---|
| Arg, % | 1.26 | 3.19 | 0.38 |
| His, % | 0.90 | 1.09 | 0.23 |
| Ile, % | 1.24 | 1.80 | 0.26 |
| Leu, % | 2.19 | 3.26 | 1.03 |
| Lys, % | 0.93 | 2.66 | 0.26 |
| Met, % | 0.27 | 0.62 | 0.19 |
| Cys, % | 0.39 | 0.68 | 0.22 |
| Phe, % | 1.44 | 2.23 | 0.43 |
| Tyr, % | 0.87 | 1.57 | 0.34 |
| Thr, % | 1.19 | 1.92 | 0.31 |
| Trp, % | / | 0.64 | 0.08 |
| Val, % | 1.58 | 1.99 | 0.40 |

Note: The data on soybean meal and corn is sourced from the "Chicken Feeding Standard" (NY/T 33-2004) of the People's Republic of China.

**Supplementary Table S2:** Microbial $\alpha$-diversity in the cecum of 21- and 42-day-old broilers

| Index type | CS | EG3 | EG6 | EG9 | EG12 | p-Value |
|---|---|---|---|---|---|---|
| 21d-sobs | 502.70±47.07 | 506.3±76.85 | 516.5±72.29 | 554.3±101.00 | 623.5±47.53 | 0.035 |
| 21d-shannon | 3.59±0.31 | 3.49±0.34 | 3.69±0.40 | 3.938±0.44 | 4.16±0.33 | 0.054 |
| 21d-simpson | 0.11±0.06 | 0.13±0.07 | 0.10±0.04 | 0.08±0.04 | 0.06±0.03 | 0.172 |
| 21d-ace | 645±85.47 | 654.3±91.38 | 650±106.1 | 683.9±122 | 776.8±58.18 | 0.063 |
| 42d-sobs | 883.2±63.03 | 711.3±90.83 | 742.5±26.88 | 719.8±38.96 | 722.3±38.24 | 0.007 |
| 42d-shannon | 4.46±0.37 | 4.44±0.22 | 4.461±0.11 | 4.42±0.21 | 4.31±0.20 | 0.530 |
| 42d-simpson | 0.05±0.03 | 0.04±0.01 | 0.03±0.01 | 0.04±0.01 | 0.05±0.02 | 0.371 |
| 42d-ace | 1114±72.32 | 930.7±148.1 | 957.4±41.72 | 952.1±67.2 | 961.5±57.07 | 0.007 |

**Supplementary Table S3:** The composition and percentage of cecal microbiota at the phylum level in broilers at 21-days-old of age.

| Phylum name | CS | EG3 | EG6 | EG9 | EG12 | p-Value | p-adjust |
|---|---|---|---|---|---|---|---|
| F_*Firmicutes* | 85.88±7.92 | 89.21±4.92 | 83.88±12.49 | 88.79±7.05 | 91.43±5.11 | 0.441 | 0.687 |
| F_*Bacteroidota* | 12.57±6.66 | 8.47±3.68 | 7.68±4.92 | 9.84±7.09 | 12.47±6.66 | 0.384 | 0.687 |
| F_*Proteobacteria* | 0.88±1.63 | 1.86±2.44 | 1.07±0.51 | 0.27±0.23 | 0.22±0.16 | 0.700 | 0.778 |

**Supplementary Table S4:** The composition and percentage of cecal microbiota at the phylum level in broilers at 42-days-old of age.

| Phylum name | CS | EG3 | EG6 | EG9 | EG12 | p-Value | p-adjust |
|---|---|---|---|---|---|---|---|

| | | | | | | | |
|---|---|---|---|---|---|---|---|
| F_*Firmicutes* | 76.49±9.64 | 86.7±4.41 | 84.34±8.56 | 83.27±7.85 | 74.97±5.93 | 0.042 | 0.138 |
| F_*Bacteroidota* | 21.23±10.33 | 22.88±5.13 | 13.65±8.13 | 8.43±0.06 | 22.88±5.13 | 0.06 | 0.16 |
| F_*Desulfobacterota* | 1.26±0.63 | 0.24±1.18 | 0.94±1.03 | 1.03±0.70 | 0.77±0.14 | 0.456 | 0.495 |
| F_*Proteobacteria* | 0.25±0.03 | 0.43±0.36 | 0.18±0.14 | 1.53±1.23 | 0.94±0.43 | 0.179 | 0.332 |

**Supplementary Table S5:** Tests for multiple differences in cecum microbial diversity in 21-day-old broilers(Kruskal-Wallis H)，Mean±Sd%

| Genus name | CS | EG3 | EG6 | EG9 | EG12 | p-Value | p-adjust |
|---|---|---|---|---|---|---|---|
| g_*Ruminococcus_torques_group* | 3.03±1.20 | 6.36±7.16 | 7.43±4.90 | 10.79±7.12 | 7.67±3.23 | 0.024 | 0.3215 |
| g_*norank_f__Eubacterium_ coprostanoligenes_group* | 0.17±0.09 | 0.88±1.32 | 1.52±0.94 | 0.92±0.39 | 1.51±0.38 | 0.006 | 0.3027 |
| g_*Colidextribacter* | 1.43±0.69 | 0.67±0.33 | 0.56±0.23 | 1.20±0.40 | 0.95±0.57 | 0.025 | 0.3215 |
| g_*GCA-900066575* | 0.51±0.38 | 0.37±0.23 | 0.20±0.15 | 0.35±0.13 | 1.44±0.61 | 0.005 | 0.3027 |

**Supplementary Table S6:** Tests for multiple differences in cecum microbial diversity in 42-day-old broilers(Kruskal-Wallis H)，Mean±Sd%

| Genus name | CS | EG3 | EG6 | EG9 | EG12 | p-Value | p-adjust |
|---|---|---|---|---|---|---|---|
| g_*Alistipes* | 16.41±10.95 | 8.19±3.58 | 9.54±4.27 | 11.94±7.98 | 20.53±5.97 | 0.036 | 0.2148 |
| g_*norank_f__norank_o _Clostridia_UCG-014* | 4.94±1.33 | 10.77±4.92 | 12.64±7.08 | 8.67±3.39 | 10.26±2.99 | 0.023 | 0.1750 |
| g_*Christensenellaceae_R-7_group* | 2.88±1.44 | 4.83±1.16 | 8.06±3.02 | 8.15±3.86 | 9.71±3.13 | 0.003 | 0.0802 |
| g__*Lactobacillus* | 7.00±8.81 | 13.09±8.85 | 6.92±4.30 | 2.13±2.15 | 3.52±2.75 | 0.024 | 0.1781 |
| g_*Ruminococcus_torques_group* | 4.98±0.98 | 4.45±2.19 | 7.42±2.17 | 5.16±2.56 | 2.64±1.20 | 0.012 | 0.1291 |
| g_*unclassified_f__Oscillospiraceae* | 3.81±3.79 | 3.81±3.79 | 3.08±1.086 | 1.26±1.52 | 1.42±0.85 | 0.021 | 0.1685 |
| g_*Lachnoclostridium* | 1.19±0.38 | 1.38±0.34 | 2.25±0.68 | 2.27±1.27 | 2.05±0.58 | 0.007 | 0.1291 |
| g_*Shuttleworthia* | 1.34±0.78 | 1.66±0.85 | 0.73±0.24 | 0.82±0.40 | 0.83±0.33 | 0.039 | 0.2195 |
| g_*norank_f__Erysipelotrichaceae* | | | | | | | |
| g_*Enterococcus* | 0.08±0.08 | 0.67±0.87 | 0.15±0.26 | 1.90±3.56 | 1.37±0.87 | 0.009 | 0.1291 |
| g_*Parabacteroides* | 0.89±0.55 | 0.83±1.55 | 1.55±2.87 | 0.09±0.07 | 0.33±0.26 | 0.011 | 0.1291 |
| g_*Colidextribacter* | 1.31±0.32 | 0.63±0.29 | 0.78±0.43 | 0.47±0.31 | 0.26±0.10 | <0.001 | 0.053 |
